# A Protocol for Rehabilitating the Bypassed Limb Prior to Reversal of Jejunoileal Bypass

**DOI:** 10.1007/s11695-021-05247-7

**Published:** 2021-02-06

**Authors:** Sara Santini, Michel Suter, Maude Martinho-Grueber, Carole Monney Chaubert, Mohammed Barigou, Lucie Favre, Peter Kopp, Anne Kouadio

**Affiliations:** 1 Division of Endocrinology, Diabetology, and Metabolism, Lausanne University Hospital, 1011 Lausanne, Switzerland; 2 Department of Visceral Surgery, Lausanne University Hospital, 1011 Lausanne, Switzerland; 3 Department of Surgery, Riviera-Chablais Hospital, 1847 Rennaz, Switzerland; 4 Faculty of Biology and Medicine, University of Lausanne, Lausanne, Switzerland; 5 Division of Gastroenterology, Bern University Hospital, 3010 Berne, Switzerland; 6 Division of Internal Medicine, North Vaud Hospital Establishments (eHnv): St Loup Hospital, 1318 Pompaples, Switzerland

## Introduction

Jejunoileal bypass (JIB), the very first bariatric procedure, creates a short-bowel syndrome with approximately 35 cm of jejunum and 15 cm of ileum and a very long excluded small bowel blind loop. It was abandoned because of deleterious or lethal side effects [1, 2]. Indications prompting JIB reversal include diarrhea, electrolyte abnormalities, malnutrition, organ failure, arthritis, and poor quality of life [1–3]. JIB reversal is usually performed using a two-stage open approach [3]: first, a jejunostomy feeding tube is placed in the excluded limb, and then, after 3–12 months, intestinal continuity is re-established.

Despite some small series [1–10, Supplementary Table], there is a lack of recommendations how to best implement (re)nutrition before JIB reversal. Previous reports provide no details on progression to half-strength nutrition, and refeeding intolerance of the excluded limb has been reported [3, 4]. Here, we propose a refeeding protocol for the excluded limb prior to restauration of bowel continuity that has been successfully applied in two patients undergoing JIB reversal.

## Case Presentation

### Patient 1

**Fig. 1:**
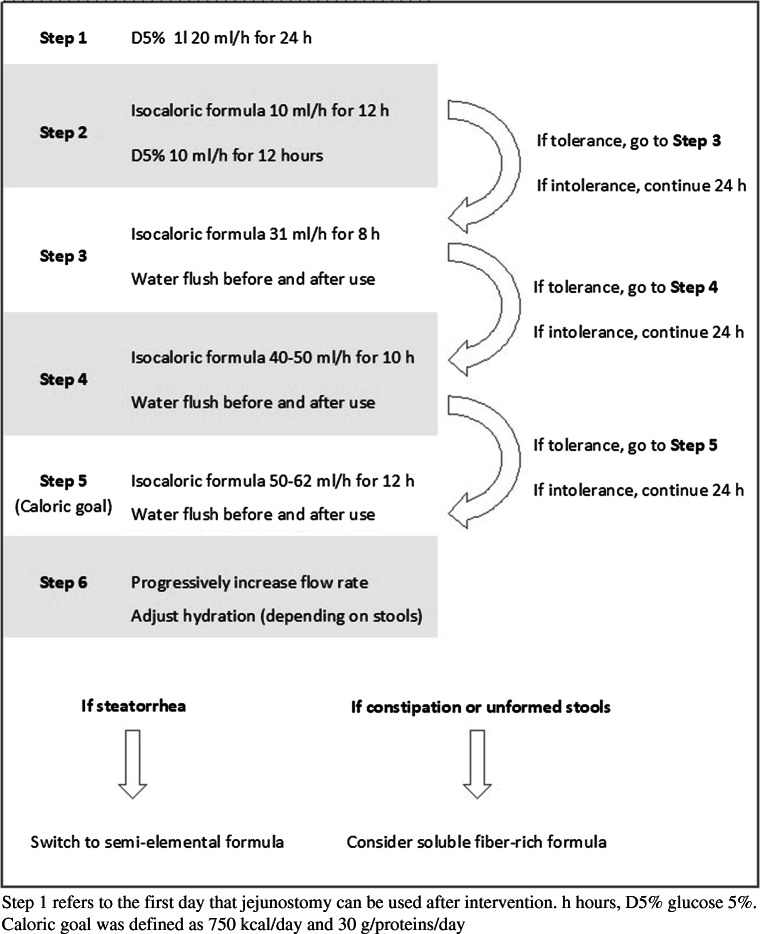
Schematic of enteral refeeding protocol via jejunostomy

A 69-year-old Caucasian female underwent JIB in 1981 (BMI = 36 kg/m^2^). Her weight dropped by 40% (60 kg, BMI = 22 kg/m^2^) and then stabilized through hyperphagia. In 2014, she was hospitalized for severe malnutrition secondary to esophageal candidiasis (additional 18% weight loss, 45 kg, BMI = 16.5 kg/m^2^). She was referred to our center in 2015 because of severe malnutrition with stage 4 chronic kidney disease with oxalic nephropathy, diarrhea, multiple electrolyte and micronutrient deficiencies, anemia, metabolic/lactic acidosis, and osteoporosis, but no evidence for liver disease. Complementary enteral nutrition via nasogastric tube was started. Despite high dose supplements of electrolytes and bicarbonate, laboratory tests did not improve sufficiently, prompting JIB reversal. After a 20% weight gain, enteral nutrition was discontinued. A jejunostomy feeding tube was placed. For rehabilitating the excluded limb, we used the refeeding protocol shown in Fig. 1. Briefly, a 5% glucose solution (D5, 20 ml/h) was administered for 24 h. The next day, an isocaloric enteral formula was administered at 10 ml/h for 12 h (Isosource standard®/Nestlé) with D5 for the remaining 12 h. After the third day, the daily amounts of formula were gradually increased (10–30 ml/h) to a total of 750 kcal/day and 29 g/day of protein.

Tolerance of the refeeding protocol via the jejunostomy was good, and the caloric goal was reached within 5 days. Because of steatorrhea, Isosource® was substituted by a semi-elemental formula (Peptamen®/Nestlé, 750 kcal/day, 30 g/day, 60 ml/h) after 1 month, with subsequent improvement of fat absorption. Four months later, she underwent open reversal of the JIB. After 1 year, quality of life and nutritional status were improved (65 kg, BMI = 23.7 kg/m^2^), kidney function stabilized (Table 1), and micronutrient supplementation was discontinued.

**Table 1:** Laboratory tests before and 3–6 months after reversal of JIB

Patient 1	Before reversal (2016)	After reversal (2018–2019)
Creatinine (44–80 μmol/l)	280	290
Corrected calcium (2.15–2.55 mmol/l)	1.75	2.4
Magnesium (0.65–1.10 mmol/l)	0.29	1.06
Bicarbonate (21.0–28.5 mmol/l)	17.4	24.9
Vitamin D (8.8–44.2 μg/l)	3.8	30
Ferritin (30–300 μg/l)	73	99
Prothrombin time (80–120%)	30	110
Prealbumin (0.20–0.40 g/l)	0.16	0.20
Albumin (35–52 g/l)	35	45
Copper (12.5–23.6 μmol/l)	3.9	20.8
Zinc (10.1–17.0 μmol/l)	6	10.6
Oxaluria (80–490 μmol/l)	1108	507
Citraturia (15–32 mg/l)	< 15	19
Patient 2	Before reversal (2017)	After reversal (2018–2019)
Creatinine (44–80 μmol/l)	150	119
Potassium (3.5–5 mmol/l)	3.1	4.6
Corrected calcium (2.15–2.55 mmol/l)	2.04	2.24
Phosphate (0.80–1.40 mmol/l)	0.70	1.24
Bicarbonate (21.0–28.5 mmol/l)	10	24
Vitamin E (11.6–41.8 μmol/l)	0.33	N/A
Vitamin A (1.05–2.09 μmol/l)	0.1	1.6
Vitamin D (8.8–44.2 μg/l)	9.1	22
Folate (8.8–60.8 nmol/l)	20	7.2
Prothrombin time (80–120%)	70	100
Hemoglobin (133–177 g/l)	97	154
Prealbumin (0.20–0.40 g/l)	0.08	0.17
Albumin (35–52 g/l)	26	44
Selenium (750–1500 nmol/l)	600	808
Copper (12.5–23.6 μmol/l)	8.2	13
Zinc (10.1–17.0 μmol/l)	6.9	10.3
Oxaluria (80–490 μmol/l)	800	142
Citraturia (15–32 mg/l)	< 15	70

*N/A* not assessed. Normal values in parenthesis

### Patient 2

A 50-year-old Caucasian male underwent JIB in 1987 (BMI = 48.8 kg/m^2^). He lost 52% of his weight and remained stable at 62 kg (BMI = 23.7 kg/m^2^) through hyperphagia. He was hospitalized in 2016 because of esophageal candidiasis with severe malnutrition (additional 27% weight loss, BMI = 15.6 kg/m^2^), diarrhea, delirium, cerebellar ataxia, and peripheral neuropathy, with multiple electrolyte and micronutrient deficiencies, stage 3B chronic kidney failure with hyperoxaluria, and metabolic acidosis (Table 1), but no sign of liver disease.

Ataxia and peripheral neuropathy were associated with copper and vitamin E deficiencies. Delirium resolved after antibiotic treatment, suggesting D-lactic acidosis and toxic bacterial overgrowth as contributing factors. Through enteral nutrition via nasogastric tube, the patient’s weight progressively increased to 62 kg (BMI = 23.7 kg/m^2^) over 15 months, but several micronutrient deficiencies and metabolic acidosis persisted, leading to the decision to perform JIB reversal. A jejunostomy was placed directly in the excluded loop. The same refeeding protocol (Fig. 1) was applied.

He tolerated the refeeding protocol similarly well and reached the caloric target (750 kcal/day, 29 g/day of protein, 62 ml/h) at day 9. Because of constipation, Isosource® was subsequently changed to a water-soluble fiber-rich formula (Novasource GI forte®/Nestlé), applied through both the nasogastric tube and the jejunostomy, resulting in better stool formation. After 3 months, improved absorptive ability of the excluded loop allowed to increase the jejunal formula (1500 kcal/day, 60 g/day of protein, 100 ml/h) and to remove the nasogastric tube. Six months later, the patient’s condition improved, ataxia resolved, and he underwent open JIB reversal. After 1 year, the patient’s weight increased (79 kg, BMI = 30 kg/m^2^), quality of life was good, and kidney function improved (Table 1).

## Discussion

These patients illustrate the well-known deleterious sequels after JIB and emphasize that reversal surgery should be considered to partially/completely correct the severe adverse effects and prevent further progression of organ failure [5, 6].

To our knowledge, this is the first description of nutritional management of JIB patients before reversal. First, the nutritional status should be improved to reduce postoperative complications, through parenteral and/or enteral nutrition according to current local guidelines on severe malnutrition. Despite severe malabsorption after JIB, enteral feeding can be effective to improve nutritional status before surgery.

Timing and proper preparation for JIB reversal is crucial, mainly due to the size discrepancy between the excluded and the functional limb. This difference is caused by the atrophy of the enteral mucosa due to prolonged exclusion of nutrients from the intestinal lumen. The two patients presented here had JIB anastomoses at about 30 cm from the angle of Treitz and 15–20 cm from the cecum, and they underwent distinct jejunostomy placement procedures. In patient 2, the excluded limb was large enough to allow for direct placement of a Witzel jejunostomy at its proximal end, while it was too narrow in patient 1. In the latter, we performed a latero-lateral anastomosis between the proximal jejunum about 20 cm from the angle of Treitz. The jejunostomy was then placed a few centimeters proximally from this anastomosis, and the enterostomy passed 30–40 cm distally into the excluded loop. This approach places the jejunostomy on non-atrophic jejunum and allows refeeding of the excluded limb. The refeeding protocol was, however, identical, well-tolerated, and effective in both patients, permitting successful restauration of bowel continuity 4–6 months after jejunostomy, comparable to other reports in the literature [3, 4].

We recommend progressive refeeding of the atrophic limb in five steps (Fig. 1). We believe that early enteral infusion of 5% glucose is safe for the extremely atrophic mucosa and is useful to test the patency of the loop after jejunostomy placement. The transition to an isocaloric formula was successful in both cases. The enteral flow rate should be gradually increased (10–20 ml/h/day) according to individual digestive tolerance. Nutrition, together with mechanical stimuli, allows to regain the integrity of the bowel after several months. The enteral absorption recovery was evident after JIB reversal in both patients who regained and stabilized their weight and corrected their micronutrient deficiencies. The two patients reported a progressive increase in their abdominal girth, although this could not be documented by objective measurements. This observation illustrates intestinal adaptation despite the extremely long mucosal inactivity.

Exclusion of the atrophic limb also affects pancreatic enzyme action resulting in steatorrhea (see Patient 1). In this case, semi-elemental formula, containing peptides and medium-chain triglycerides, should be tested. Moreover, hydration of the atrophic limb must be adapted to prevent digestive transit disorders.

In summary, this progressive refeeding protocol of the excluded limb via feeding enterostomy prior to surgery is an essential component for successful JIB reversal.

## Supplementary Information

ESM 1 (DOCX 17.2 kb)
