# Supplementary material for: A Protocol for Rehabilitating the Bypassed Limb Prior to Reversal of Jejunoileal Bypass
Source: Obes Surg. 2021 Feb 6;31(6):2776–9. doi: 10.1007/s11695-021-05247-7 (PMC8113190; doi:10.1007/s11695-021-05247-7)
Supplement: Supplementary file 1 — (DOCX 17.2 kb) [file 11695_2021_5247_MOESM1_ESM.docx]

**Supplementary Table.** Synopsis of JIB reversals reported between 2000 and 2020.

| **Authors** | **Number of patients** | **Indication for reversal** | **Laparotomy versus laparoscopic approach** | **Refeeding protocol via jejunostomy** | **Refeeding tolerance** | **Additional intervention** | **Clinical outcomes**  **after reversal** | **Further**  **surgery** |
| --- | --- | --- | --- | --- | --- | --- | --- | --- |
| Pelà et al.  2018 [6] | 1 | Heart and liver failure, malnutrition, electrolyte imbalance | Laparotomy | N/A | N/A | Electrolyte and micronutrient supplementation | Improvement of hepatic and cardiac function | No |
| Justice et al.  2018 [4] | 1 | Oxalate nephropathy, enteritis, malnutrition | Laparoscopic with conversion to laparotomy | Initially elemental half strength nutrition 35 ml/h for 12h/day, followed by full-strength formula for 5 h/day during a few weeks | Poor | Parenteral nutritional support, micronutrient supplementation | Weight loss,  stable renal function, functional bowel obstruction | Yes |
| Ardila-Gatas J. et al.  2016 [7] | 1 | Diarrhea, malnutrition,  oxalate nephropathy | Laparoscopic | N/A | N/A | No | Weight gain, resolution of diarrhea | No |
| Chousleb et al.  2012 [2] | 4 | Diarrhea, malnutrition,  oxalate nephropathy | Laparotomy | N/A | N/A | N/A | Weight gain,  improvement of life quality | No |
| Brolin E. et al.  2009 [1] | 3 | Diarrhea, electrolyte imbalance | Laparotomy | N/A | N/A | N/A | Weight gain | No |
| Lee H et al.  2009 [8] | 1 | Diarrhea, osteoporosis, dermatitis, arthralgia, oxalate kidney stones, malnutrition, BBS | Laparotomy | N/A | N/A | Sleeve gastrectomy,  micronutrient and protein supplementation | N/A | No |
| Patel et al.  2008 [9] | 1 | Malnutrition, oxalate nephropathy, diarrhea, liver failure, chronic pancreatitis, peripheral neuropathy | Laparotomy | N/A | N/A | No | Weight gain,  Improvement of hepatic and renal function | Sleeve gastrectomy |
| Dallal et al.  2006 [3] | 1 | Enteritis, arthritis, dermatitis, malnutrition | Laparoscopic | Half-strength Gatorade solution, followed by full-strength Ensure during 3 months | Poor | Metronidazole | Weight gain, resolution of enteritis, dermatitis | No |
| Dhar et al.  2005 [5] | 4 | Oxalate nephropathy | Laparotomy | N/A | N/A | Alkalization therapy | Normalization of oxaluria | No |
| Vage et al.  2002 [10] | 10 | Malnutrition, oxalate nephropathy, diarrhea, liver failure, peripheral neuropathy | Laparotomy | N/A | N/A | N/A | Weight gain, resolution of peripheral neuropathy, improvement of liver function | Gastric banding  (3 patients) |

N/A: Non assessed. BBS: Brown bowel syndrome (deposition of lipofuscin in intestinal muscularis propria).
